# New Anti-HBV C-Boivinopyranosyl Flavones from *Alternanthera philoxeroides*

**DOI:** 10.3390/molecules21030336

**Published:** 2016-03-14

**Authors:** Bin Li, Qing-Lan Guo, Ying Tian, Shi-Jun Liu, Qiong Wang, Li Chen, Jun-Xing Dong

**Affiliations:** 1 Department of Pharmaceutical Chemistry, Beijing Institute of Radiation Medicine, Beijing 100850, China; Jkylibin@hotmail.com (B.L.); Hq6106@gmail.com (Y.T.); lsj2518@sina.com (S.-J.L.); wqwangqiongwq@163.com (Q.W.); hpch2003@gmail.com (L.C.); 2 Institute of Materia Medica, Chinese Academy of Medical Sciences and Peking Union Medical College, Beijing 100050, China; guonina@imm.ac.cn

**Keywords:** *C*-boivinopyranosyl flavones, *Alternanthera philoxeroides*, anti-HBV activity

## Abstract

*C-*boivinopyranosyl flavones have rarely been isolated from nature. In the search for anti-HBV (hepatitis b virus) constituents of *Alternanthera philoxeroides*, two new compounds, luteolin-6-*C*-β-d-boivinopyranosyl-3′-*O*-β-d-glucopyranoside (**1**) and chrysoeriol-6-*C*-β-d-boivinopyranosyl-4′-*O*-β-d-glucopyranoside (**2**), along with three known *C*-boivinopyranosyl flavones (compounds **3**–**5**) were isolated. Their structures were determined by spectroscopic analyses including 1D and 2D NMR, HR-ESI-MS, IR spectra. Compounds **1**, **2** and **3** showed significant anti-HBV activities through specifically inhibiting the secretion of HBsAg in HepG2.2.15.

## 1. Introduction

It is estimated that approximately 350 million people worldwide and 93 million in China alone are hepatitis b virus (HBV) carriers, causing approximately 500,000 deaths every year.

The current treatment strategies involving vaccines, interferons and nucleosides are unsatisfactory due to drug-resistance and adverse side effects [1,2,3].

For many years, herbs have been used as the Traditional Chinese Medicine for treatment of hepatitis B virus infection, such as *Salvia miltiorrhiza*, *Rheum palmatum*, *Phyllanthi urinariae* [4], *etc.* In recent years, many agents derived from botanical origin have been reported to possess anti-HBV activities, such as niranthin and nirtetralin from *Phyllanthus*, curcumin from *Curcuma*, alisol A from *Alisma orientalis*, oxymatrine from *Sophora*, *etc.* [5]. Botanical agents are attractive sources of new anti-HBV drugs.

**Figure 1:**
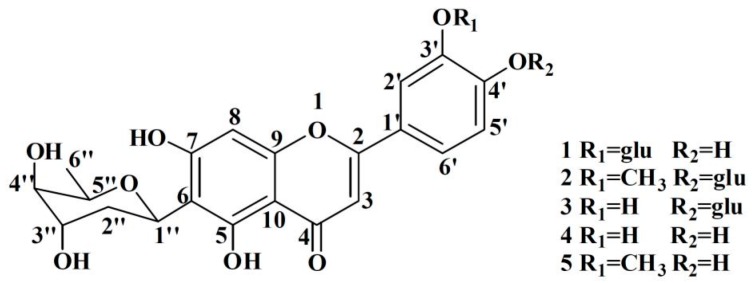
The structures of compounds **1**–**5**.

*Alternanthera philoxeroides* is widely distributed in South China and mainly used in China for the treatment of measles, influenza, encephalitis b. Its major constituents are triterpenoid saponins, flavones, phytosterols, anthraquinones and organic acids [6]. Several oleanolic acid analogues from it were reported to possess anti-HBV activities. In this paper, two new 6-*C*-boivinopyranosyl flavones together with three known analogues were isolated from *Alternanthera philoxeroides* (Figure 1), and their anti-HBV activities were evaluated toward HepG2 2.2.15 cells.

## 2. Results and Discussion

**Figure 2:**
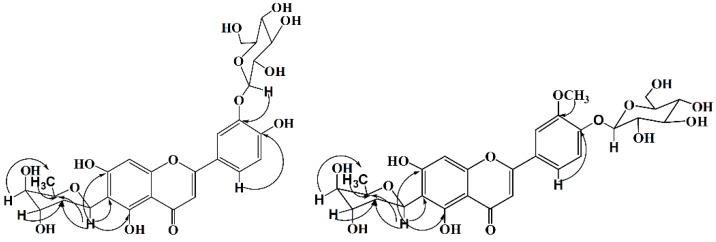
Key HMBC correlations of compounds **1** and **2**.

Compound **1** was obtained as a yellow amorphous powder. HR-ESI-MS *m*/*z* 579.1728 [M + H]^+^ (calcd for C_27_H_31_O_14_, 579.1708) showed the molecular formula C_27_H_30_O_14_. IR spectrum indicated the presence of hydroxyl (3447 cm^−1^), carbonyl (1652 cm^−1^) and phenyl (1574, 1495 cm^−1^) groups in the molecule. Fifteen carbon signals in low field of ^13^C-NMR similar to those of luteolin, together with δ 6.83 (1H, s), 6.58 (1H, s), δ 7.79 (1H, d, *J* = 1.8 Hz), 6.96 (1H, d, *J* = 8.4 Hz) and 7.65 (1H, dd, *J* = 1.8, 8.4 Hz) in ^1^H-NMR showed an 6, 3′ (or 6, 4′) disubstituted luteolin structure (Table 1). According to the HMQC and HMBC spectra, the NMR chemical shifts of the carbons signals could be assigned unambiguously. The HMBC correlations indicated a 2,6-dideoxygenated pyranosyl directly linked to C-6, which could be identified as boivinopyranosyl by small coupling constants of 3′-H (br.s) and 4′-H (br.s). The connectivity of C3′-O-C1′′′ was deduced by the HMBC correlation of H-1′′′/C-3′ (Figure 2). A β-d-glucopyranosyl was recognized by the anomeric proton signal at 4.89 (d, *J* = 7.2 Hz) in the ^1^H-NMR spectrum and also by the characteristic six signals at δ 101.9 (CH), 73.3 (CH), 75.9 (CH), 70.1 (CH), 77.4 (CH), and 60.9 (CH_2_) in the ^13^C-NMR spectrum (Table 1). This conclusion was confirmed by comparing acid-hydrolyzed (2N-HCl, 80 °C, 3 h) product of 1 with an authentic d-glucopyranose sample on Si TLC (R*_f_* = 0.4). Therefore, compound **1** was established as luteolin-6-*C*-β-d-boivinopyranosyl-3′-*O*-β-d-glucopyranoside.

Compound **2** was obtained as a yellow amorphous powder. HR-ESI-MS *m*/*z* 593.1858 [M + H]^+^ (calcd for C_28_H_33_O_14_, 593.1865) showed the molecular formula C_28_H_32_O_14_. IR spectrum indicated the presence of hydroxyl group (3395 cm^−1^), carbonyl group (1655 cm^−1^) and phenyl group (1591, 1492 cm^−1^) in the molecule. NMR data (Table 1) similar to those of **1** indicated a 6-*C*-β-d-boivinopyranosyl flavone glucoside. HMBC correlations proved the locations of -OCH_3_ at C-3′ and glucopyranosyl at C-4′ (Figure 2). Consequently, the structure of compound **2** was elucidated as chrysoeriol-6-*C*-β-d-Boivinopyranosyl-4′-*O*-β-d-glucopyranoside.

Three known 6-*C*-d-boivinopyranosyl flavones were isolated and their structures were identified as luteolin-6-*C*-β-d-boivinopyranosyl-4′-*O*-β-d-glucopyranoside (**3**) [7], luteolin-6-*C*-β-d-boivinopyranoside (**4**) [8], chrysoeriol-6-*C*-β-d-boivinopyranoside (**5**) [9] by comparison of their spectroscopic data with those reported in the literature.

These flavones were tested for their potential anti-HBV activities according to inhibitory secretion of HBV surface antigen (HBsAg) and HBV e antigen (HBeAg) in HBV-infected HepG2.2.15 under non-cytotoxic concentration and the results were summarized in Table 2. Compounds **1**, **2**, and **3** significantly blocked the secretion of HBsAg in a dose dependent manner.

## 3. Experimental

### 3.1. General Experimental Procedures

^1^H- and ^13^C-NMR spectra were obtained on a Bruker ECA-400 MHz (Billerica, MA, US) and a Varian UNITYINOVA 600 (Salt Lake City, UT, USA) with TMS as an internal standard. IR spectra were measured on a Bruker Vertex 70. HR-ESI-MS spectra were measured on a 9.4 T Q-FT-MS Apex Qe (Bruker Co.). ESI-MS spectra were measured on a Thermo Finnigan LCQ DECA spectrometer (Madison, WI, USA). Macro porous resin AB-8 (NanKai College Chemical Inc., Tianjin, China), Silica gel (60–120 mesh, 200–300 mesh, Qingdao Marine Chemical Group Co., Qingdao, China), and Sephadex LH-20 (Pharmacia, Uppsala, Sweden) were employed for column chromatography. TLC was carried out using silica gel 60 (>230 mesh, Qingdao Marine Chemical Group Co.) and GF_254_ plates precoated with silica gel 60.

### 3.2. Plant Materials

*Alternanthera philoxeroides* was bought from Qixin decoction pieces Co.Ltd, Hebei province, China and identified by Bin Li, Department of Pharmaceutical Chemistry, Beijing Institute of Radiation Medicine.

### 3.3. Extraction and Isolation

The dried and powdered material (20 kg) was extracted with 90% ethanol for two times under reflux. The concentrated extract (2.4 kg) was suspended in water and then partitioned with petroleum ether, chloroform, EtOAc, and n-BuOH successively. The *n*-BuOH extract (400 g) was subsequently separated into five fractions by macro porous resin AB-8. Among them, 50% ethanol fraction (60 g) was subjected to silica gel column chromatography (CC) eluted with CHCl_3_/MeOH gradient (from 10:1 to 0:1) to yield five fractions A–E. Fraction A eluted with CHCl_3_/MeOH gradient (from 10:1 to 3:1) on silica gel again to yield fractions A1 and A2. They were chromatographed respectively by Sephadex LH-20 (with CHCl_3_/MeOH, 1:1) to obtain compound **1** (58 mg), and compound **3** (63 mg). Fraction B eluted with CHCl_3_/MeOH gradient (from 10:1 to 5:1) on silica gel again to yield fractions B1, B2 and B3. They were chromatographed respectively by Sephadex LH-20 (with CHCl_3_/MeOH, 1:1) to obtain compound **2** (65 mg), compound **4** (15 mg) and compound **5** (85 mg).

Compound **1**: yellow amorphous powder. IR (KBr) ν_max_ 3447, 1652, 1574, 1495 cm^−1^; HR-ESI-MS *m*/*z* 579.1728 [M + H]^+^ (calcd for C_27_H_31_O_14_, 579.1708); ^1^H-NMR (DMSO-*d*_6_, 400 MHz) and ^13^C-NMR (DMSO-*d*_6_, 100 MHz) spectroscopic data, see Table 1.

Compound **2**: yellow amorphous powder.IR (KBr) ν_max_ 3395, 1655, 1591, 1492 cm^−1^; HR-ESI-MS *m*/*z* 593.1858 [M + H]^+^ (calcd for C_28_H_33_O_14_, 593.1865); ^1^H-NMR (DMSO-*d*_6_, 400 MHz) and ^13^C-NMR (DMSO-*d*_6_, 100 MHz) spectroscopic data, see Table 1.

### 3.4. Anti-HBV Assay

The anti-HBV assay was performed according to the previous report [10]. The inhibition of the secretions of HBsAg and HBeAg was assayed by ELISA method; and the cytotoxicity was assessed by the MTT method.

#### 3.4.1. Inhibition Assay of HBsAg and HBeAg Secretions HepG

The HepG2.2.15 cells were plated in 96-well cell plates at a density of 5 × 10^4^ cells·mL^−1^ in 200 μL of DMEM medium, and routinely cultured at 37 °C under 5% CO_2_. Different concentrations of the studied compounds were supplemented to the medium after cells were plated. Control cultures received the carrier solvent (DMEM with 0.2% DMSO). Cells were grown in the presence of the studied compounds for 8 days with changing the medium on the 4th day. The suspension and the cells were separated and collected for HBsAg and HBeAg level tests immediately. The inhibiting rates (%) were calculated by comparing the treatment group with the tested compounds and the solvent control group with DMSO. The percent of inhibition (%) = [1 − OD value of sample well/OD value of DMSO well] × 100.

#### 3.4.2. MTT-Based Cytotoxicity Assay

HepG2.2.15 cells were cultured with test compounds for 8 days. After cultivation, cell proliferation was determined by MTT assay. Briefly, 10 μL of MTT (5 g·mL^−1^) was added to each well and further incubated for 4 h. Then, the culture medium was removed from each well and DMSO was added to dissolve the purple formazan of MTT. The absorbance at 540 nm was read in the Multiskan MK3 (Thermo).

## 4. Conclusions

*C*-boivinopyranosyl flavones have rarely been isolated from nature; fewer than 20 compounds could be found by SCifinder. In this article, two new *C*-boivinopyranosyl flavones, along with three known compounds were isolated and identified. Compounds **1**, **2**, and **3** significantly blocked the secretion of HBsAg in a dose dependent manner. They inhibited HBsAg secretion respectively by 70.6% (compound **1**), 74.1% (compound **2**) and 67.3% (compound **3**) at non-cytotoxic concentration of 129 μM (compounds **1** and **3**), 127 μM (compound **2**).

## Figures and Tables

**Table 1:** ^1^H-NMR (400 MHz) and ^13^C-NMR (100 MHz) data for compound **1** and **2** in DMSO-*d*_6_.

Position	Compound 1		Compound 2	
	δ_H_	δ_C_	δ_H_	δ_C_
2		163.5		163.2
3	6.83 (s)	103.4	7.03 (s)	104.0
4		182.0		182.1
5		157.1		157.2
6		110.4		110.4
7		162.5		162.6
8	6.58 (s)	94.8	6.63 (s)	95.0
9		156.1		156.2
10		103.2		103.5
1′		121.2		123.9
2′	7.79 (d, *J =* 1.8 Hz)	114.4	7.63 (d, *J =* 2.4 Hz)	110.2
3′		145.6		149.8
4′		150.7		149.2
5′	6.96 (d, *J* = 8.4 Hz)	121.9	7.25 (d, *J =* 8.8 Hz)	115.0
6′	7.65 (dd, *J* = 1.8, 8.4 Hz)	116.4	7.66 (dd, *J =* 2.4, 8.8 Hz)	120.5
boivinose				
1′′	5.30 (dd, *J =* 2.7, 12.3 Hz)	67.3	5.34 (dd, *J* = 2.8, 12.4 Hz)	67.4
2′′	1.48 (d, *J =* 13.8 Hz)	31.4	1.50 (d, *J =* 14.0 Hz)	31.4
	2.19 (ddd, *J =* 2.7, 4.2, 13.8 Hz)		2.21 (ddd, *J* = 2.4, 3.2, 14.0 Hz)	
3′′	3.83 (br.s)	66.5	3.85 (br.s)	66.5
4′′	3.23 (br.s)	68.6	3.25 (br.s)	68.7
5′′	4.02 (q, *J =* 6.6 Hz)	70.6	3.90 (q, *J* = 6.0 Hz)	70.6
6′′	1.14 (d, *J =* 6.6 Hz)	17.1	1.16 (d, *J =* 6.0 Hz)	17.1
glucose				
1′′′	4.89 (d, *J =* 7.2 Hz)	101.9	5.08 (d, *J =* 7.2 Hz)	99.5
2′′′	3.32 (m)	73.3	3.33 (m)	73.1
3′′′	3.30 (m)	75.9	3.30 (m)	76.8
4′′′	3.15 (m)	70.1	3.17 (m)	69.6
5′′′	3.47 (m)	77.4	3.36 (m)	77.1
6′′′	3.77 (m)	60.9	3.47 (m)	60.6
	3.48 (m)		3.68 (m)	
-OCH_3_			3.90 (s)	56

**Table 2:** Anti- hepatitis b virus (HBV) activity and cell proliferation of five compounds.

Compound	IC_50_ (μM)		CC_50_ (μM)
	HBsAg	HBeAg	
**1**	28.65	NE	>519
**2**	22.20	NE	>253
**3**	31.54	NE	>519
**4**	11.39	39.78	60.10
**5**	NE	NE	<21.81

IC_50_, concentration of 50% inhibition of viral antigen expression; CC_50_, 50% cytotoxic concentration; NE, no effect.
